# Revisiting Current Strategies for Primary Prevention of Motorcycle Collisions in Jamaica

**DOI:** 10.5249/jivr.v2i1.13

**Published:** 2010-01

**Authors:** Shamir O Cawich, Hyacinth E Harding, Necia R Evans, Ivor W Crandon, Allie Martin

**Affiliations:** ^*a*^Department of Surgery, Radiology, Anaesthesia and Intensive Care, The University of the West Indies, Mona, Kingston 7, Jamaica, West Indies.

## Abstract

**Abstract:**

Motorcycle Road Traffic Collisions place a heavy burden on emergency medical services in Jamaica. We explore the existing strategies and legislative policies that may prevent or reduce the severity of these injuries in Jamaica. This is an important aspect of health care as it may minimize the impact of these preventable injuries on the limited resources of the health services.

## Introduction

Jamaica is a developing Caribbean country that deals with epidemic levels of trauma.^1^ In this setting, the government funded public health care system is seriously burdened by injuries.^1-3^ Preventative strategies are important to minimize the impact of these injuries on precious health and human resources.

Road traffic collisions are the second commonest reason for trauma admissions in Jamaica, accounting for 20% of general trauma admissions at tertiary referral hospitals in Kingston.^1-4^  In this setting, 12.6% of all road traffic collision victims are injured on motorcycles^1^ and the number has been increasing over the past decade.^5^ 

Motorcycle collision victims are exposed to a greater degree of body trauma than automobile occupants involved in collisions of similar velocities because they are disadvantaged by the lack of available safety equipment such as airbags and seatbelts.^5-10^ Therefore, special consideration must be given to preventative strategies for motorcycle collisions since their poly-traumatized victims place inordinately high demands on the health care resources in this setting.^1-5^

Generally, two approaches to injury prevention are recognized: Primary prevention aims to reduce the incidence of injuries by enforcement of legislative policies that prevent collisions from occurring. The aim of secondary prevention is to limit injury severity once a collision has occurred. We explore the existing legislative policies that aim at primary prevention of these injures in Jamaica.

**Strategies for Primary Prevention:**

The government of Jamaica published a revised Road Traffic Act in 1999 that addressed the requirements for safe operation of motorcycles on Jamaican roadways.^11^  The Government of Jamaica has charged the Island Traffic Authority with enforcement of the Road Traffic Act.^11^  The Road Traffic Act addressed motorcycle use in several clauses:

**1- Motorcycle Roadworthiness: Lights, Warning Devices, Horns**

No motor vehicles are licensed to be “used on Jamaican roads unless there has been issued a certificate of fitness” and the “vehicle has been issued a license by the traffic authority” (Section 10). Prior to receiving a certificate of fitness, vehicles must be inspected by the Traffic Authority “to ensure their worthiness for use” (Section 5d).

Specifically with respect to motorcycles, this requires that motorcycles be outfitted with current registration plates (Section 14), a white headlamp and red tail lamp to signal the approach and position of the motorcycle (Section 40-2), an audible horn to warn of motorcycle’s approach (Section 43-1) and functional braking mechanisms (Section 43-2). Any person found operating a motorcycle in contravention of these regulations is considered guilty of a punishable offence (Section 43-5).

Compared to traditional automobiles, motorcycles are smaller and easier to maneuver, allowing riders to effortlessly negotiate other motor vehicles in traffic. These riders are at an increased collision risk because motorcycles are less conspicuous to automobile drivers.^6,12^ Therefore, these provisions of the Road Traffic Act promoting proper visibility and warning devices cannot be over emphasized.

**2- Driver Licensing: prerequisites, disqualifications, cancellations**

The Island Traffic Authority also has the duty of testing driver license applicants for their competence to drive (Section 5). The prerequisites for being granted a motorcycle license include “age of at least 17 years” (Section 18-4), “the ability to read and write in English” (Section 18-2), a passing mark or written exemption from a drivers test (Section 18-3), a certificate from a Justice of the Peace or Police Superintendent certifying the applicant to be “fit and proper to hold a driver’s license” (Section 18-5) and a “medical certificate from a registered practitioner certifying that he is not suffering from any disease or disability that would be likely to cause the driving by him to be a source of danger to the public” (Section 18-6). The operation of a motorcycle on Jamaican roads without a valid driver’s license is considered a punishable offence (Sect 16-1).

It has been previously documented that as motorcycle riders increased in age and maturity, the level of compliance with the provisions of the Jamaica Road Traffic Act increased.^13^ Young persons in the third and fourth decades were the individuals who were less compliant with the Traffic Act.^13^  This may have important implications on the issuance of driver licenses only to select individuals who are mature enough to handle the responsibility of motorcycle use. Although controversial, this suggestion may warrant consideration and review by legislators.

**3- Definition of traffic offences: speed limits, reckless driving, pillion riding**

Although it remains controversial, it has been suggested that many motorcycle drivers have irresponsible driving practices that contribute to the increased collision risk.^6,12^Several provisions in the Road Traffic Act^11^ promote responsible driving practices.

The Road Traffic Act^11^  has defined several punishable offences that include: Driving in excess of the maximum speed limit as specified for individualized roadways dependent on road conditions (Section 26-1); Reckless driving is defined as operating the motorcycle “at a speed or in a manner which is dangerous to the public, having regard to the nature, condition and use of the road and the amount of traffic at the time” (Section 27-1); And driving without due care and attention is defined as “utilizing public roadways without reasonable consideration for others using the road” (Section 32-1).

It has previously been documented that pillion passengers have the highest levels of non-compliance with the Road Traffic Act.^13^ The Road Traffic Act limits transport to “only one pillion passenger on any two wheeled motorcycle” (Section 35-1) and requires that they be carried “sitting astride the cycle and on a proper seat securely fixed to the cycle behind the drivers seat” (Section 35-1).

We believe that closer attention needs to be placed on the “bike back culture” that has been described in this setting where pillion passengers (usually females) are scantily clad to maximize their body exposure, without protective garments or helmets.^13^ The legislators should pay closer attention to this practice as the unprotected pillion passengers place themselves at risk by engaging in this practice. Additionally, this can be a very distracting practice to automobile drivers also utilizing the roadways, thereby increasing the likelihood of collisions. The Traffic Authority should launch educational campaigns to educate pillion passengers about their risk and to ensure that they do not misinterpret provisions of the Road Traffic Act as only being applicable to drivers.

**4- Alcohol impaired driving**

Alcohol impaired driving is a problem that has been singled out because it has been identified as an important cause of road traffic morbidity and mortality worldwide.^14^ The National Road Safety Council in Jamaica reported that 3.3% of all road traffic collisions between 2000 and 2003 were attributable to the influence of alcohol^15^ but there is no further specific information on alcohol related motorcycle collisions in Jamaica.

Alcohol has several deleterious effects, including impaired behavioral and cognitive capacities,^14,16^ reduced reaction time,^14,17,18^ impaired judgment^14,15,17,19^  and reduced visual acuity.^17,18,19,20^ Inebriated drivers are also less likely to use protective devices and more likely to exceed speed limits.^21,18^

This is a complicated problem that encompasses several social and public health issues. Several different countermeasures have already been implemented in Jamaica, including public education campaigns and legislation. The Road Traffic Act (Section 34G1) defines the legal limit for alcohol as levels being in excess of 35micrograms/100ml on breath testing and 80mg/100ml of blood.^11^ Driving under the influence of alcohol (DUI) is a punishable offence that is defined in the Road Traffic Act (Section 34-1) as an “attempt to drive a motor vehicle in a public place under the influence of drink or a drug to such extent as to be incapable of having proper control of the vehicle”.^11^

The definition of legal limits is an important step toward curbing DUI offences, but there also needs to be visible and consistent law enforcement by traffic police. Currently, any member of the Jamaica Constabulary Force has the authority to conduct breath tests on drivers suspected to have alcohol levels in excess of legal limits (Section 34B-1). Non-compliant drivers can be arrested without a warrant and, if convicted, will face monetary penalties and/or a period of imprisonment (Section 34b-5).^11^ The actual intention of enforcement activities should not be to apprehend as many offenders as possible, but rather to create the perception that those who break the law will be caught and punished.^14^Therefore, enforcement activity should be high profile and visible, including strategies such as random breath testing, sobriety check points by special DUI task forces with proper equipment and training.^14,22^ Additionally, the enforcement activities should target specific groups / individuals.^16^ International research has shown that recidivist DUI offenders are less receptive to traditional sanctions and require intense, tailored rehabilitation because many of them are alcohol abusers.^23-25^

The Jamaican government has also implemented several related pieces of legislation that restrict access to alcohol: The Child Care and Protection Act ^26^ restricts alcohol access to anyone below the legal purchasing age of 18 years (Section 62-b); the Spirit License Act^27^ regulates points of sale by requiring proprietors to be licensed to sell alcohol (Section 3); the Excise Duty Act^28^ regulates points of sale (Section 31-39); and the Customs Act^30^ imposes special taxation for alcohol purchases. Other strategies that are not yet in existence in Jamaica, but have been implemented in high income countries include the definition of lower legal limits for novice drivers, younger drivers and drivers of commercial vehicles.^14,26^

**Penalties for Offences**

Currently, the laws of Jamaica stipulate that person found to be in contravention of the Road Traffic Act is guilty of an offence punishable according to existing laws.^11^ There are two types of penalties imposed on offenders:

**1- Imprisonment / Monetary Penalties**

Any person found “liable on a conviction before a resident magistrate” is subject to a monetary fine and/or a period of imprisonment in keeping with the laws of Jamaica.^11^ There are pre-determined penalties for each listed offence that include:

-Operating a motorcycle without a valid driver’s license (Sect 16-1): Imprisonment for a term not exceeding six months or a penalty not exceeding $10,000.00 ($124.51 US).

-Operating a motorcycle without a certificate of fitness, current registration and/or motorcycle vehicle license (Section 43-5): Liable to a penalty not exceeding $3,000.00 (equivalent to $37.35 US).

-Operating a motorcycle using a provisional driver’s license without appropriate supervision (Section 16-6): Liable to a fine not exceeding $4,000 ($49.81 US).

-Operating a motorcycle without approved protective headgear (Section 43-D): Liable to a fine not exceeding $2,000.00 ($27.34 US) in the case of a first offence, and a fine not exceeding $5,000.00 ($68.35 US) in the case of a second offence.

-Operating a motorcycle on a specified road in excess of the speed limits (Section 26-1): Liable to a penalty not exceeding $2,000.00 ($27.34 US) if the maximum speed is exceeded by 10-20 miles per hour; $4,000.00 ($49.81 US) if the limit is exceeded by 21-30 mph; and $6,000.00 ($74.71 US) if the limit is exceeded by >31 mph.

-Driving a motorcycle on a public road recklessly or at a speed or in a manner that is dangerous to the public (Section 27-1): Liable to imprisonment for a term not exceeding 6 months or a penalty not exceeding $20,000.00 ($249.02 US) in the case of the first conviction; and $30,000.00 ($373.53 US) for subsequent convictions.

-Driving “on a public road without due care and attention” (Section 32-1): Liable to a penalty not exceeding $5,000.00 ($62.26 US).

-Driving “under the influence of alcohol or drugs” (Section 34-1): Liable to imprisonment for a term not exceeding 4 months (6 months for a second conviction) or a penalty not exceeding $20,000.00 ($249.02 US) and a period of disqualification from holding or obtaining a license for 12 months from the date of conviction.

-Improper transport of pillion passengers on a two wheeled motorcycle (Section 35-1): The driver shall be liable to a penalty not exceeding $2,000.00 ($24.90 US) in the case of the first conviction; and a penalty not exceeding $4,000.00 ($49.81 US) for a second conviction.

-These monetary penalties are deterrents to non-compliance with the Road Traffic Act, but these fines are not appropriate in modern times. Implementation of more realistic penalties may motivate more motorcycle users to be compliant with the helmet laws. We believe that it is time for legislators to revise the penalties for offences in Jamaica.

**2- Demerit Point System**

The Island Traffic Authority also manages a “demerit point system” and maintains records of endorsements on drivers’ licenses (Section 5). In this system, “demerit points are given by the courts in addition to any penalty for that offence” against the Road Traffic Act.^11^ The appropriate demerit points are assigned to offenders’ driver licenses by the courts and the offenders can have their license “disqualified in accordance with the demerit points accumulated” (Section 5).

The offenders can be disqualified for a period of 6 months if they have accumulated 10-14 points, one year with 15-20 points and 2 years with >20 demerit points.^11^ On a second conviction in contravention of the road traffic act, the court can exercise the power to permanently disqualify the offender from obtaining a drivers license “unless the court for any special reason thinks fit otherwise” (Section 27-3).

-While this demerit system provides an additional deterrent to non-compliant motorcyclists, it does not compensate for inappropriate monetary penalties. Additionally, both strategies are heavily dependent upon visible and consistent enforcement that have been documented to be lacking in many low income countries ^7,8,32^and in Jamaica.^12,13^

**Educational Campaigns**

Educational campaigns may be one way to increase rider compliance with traffic laws and safety precautions while promoting good driving practices.^7,8,10,12^ Campaign messages can be transmitted via mass media targeted at high risk populations. Young males are commonly thought to be the gender more involved in risk taking behavior.^6,9^ Observati-onal studies of non-compliant motorcycle collision victims revealed that most motorcycle collision victims who were non-compliant with safety precautions on Jamaican roadways were women (51% vs 15%) and pillion passengers (89% vs 46%) between the ages of 20 and 39 years.^13^Educational campaigns should target all motorcycle riders, but should pay special attention to these groups.

Several reasons for non-compliance were identified in these reports, including non-expectation of collision involvement, short distance travel, inconvenience / high cost of helmets, misinterpretation of the laws as being applicable to drivers only and the “bike back” mentality.^13^ Other authors have suggested that ignorance, cultural dispositions toward lawlessness, fatalism, insufficient educational campaigns, and/or recreational drug use are additional factors associated with non-compliance.^6,9,33^ These are all points that can be targeted by educational campaigns.

Other goals for educational campaigns could include increasing driver awareness about the dangers of impaired driving, ensuring that they are informed about existing laws, the risk of being detected, and the legal consequences of this behavior. These are important elements of any approach toward reducing alcohol-related collisions.^16^

Although it has not been discussed here, the importance of secondary preventative strategies that aim to limit tissue damage once a collision has occurred cannot be underscored. Legislative policies to limit injury severity once a collision has occurred are already in existence in Jamaica.^11,13^ However, enforcement remains a challenge in this resource strapped setting.^13^ Public educational campaigns and visible law enforcement should prevent or reduce the severity of injuries consequent to motorcycle collisions on Jamaican roads.

## Conclusions

It is time for the country to re-examine the current primary preventative strategies to prevent motorcycle collisions. This situation is dire, especially in the current economic climate where there is need to preserve precious health and human resources that are spent as a result of these incidents.

We must focus on educational campaigns for good driving practices as well as visible and consistent enforcement of road traffic laws. Finally, it is also time for legislators to re-examine the existing laws to ensure that the penalties for non-compliance are applicable to this modern era.

## References

[1] Crandon I, Carpenter R, McDonald A (1994). Admissions for trauma at the University Hospital of the West Indies.A prospective study. West Ind Med J.

[2] Scarlett MD, Mitchell VT, Amata AO (2001). Trauma admissions to the ICU of University Hospital of the West Indies, Kingston, Jamaica. Trauma Care.

[3] Williams EW, Reid M, Lindo JLM, Williams-Johnson J, French S, Singh P (2007). Association between exposure / non-exposure to the mandatory seat belt law with regards to compliance in vehicle accident victims – a hospital review. West Ind Med J.

[4] Escoffery CT, Shirley SE (2002). Traumatic deaths in Jamaica: a coroner's autopsy study from the University Hospital of the West Indies. Med Sci Law.

[5] Crandon IW, Harding HE, Cawich SO, McDonald AH, Fearron-Boothe D. Motorcycle accident injury profiles in Jamaica: An audit from the University Hospital of the West Indies. Int J Injury Cont Saf Prom. 2009; In press. 10.1080/17457300903024236 19941216

[6] Hurt HH, Ouellet JV, Thom DR. Motorcycle Accident Cause Factors and Identification of Countermeasures, Traffic Safety Center. University of Southern California. Available at: Uhttp.www.clarity.net/%7Eadam/hurt-report.htmlU, accessed August 31, 2008.

[7] Solagberu BA, Ofoegbu CKP, Nasir AA, Ogundipe OK,  Adekanye AO, Abdur-Rahman  L (2006). Motorcycle injuries in a developing country and the vulnerability of riders, passengers, and pedestrians. Inj Prevent.

[8] Forjuoh SN (2003). Traffic-related injury prevention interventions for low-income countries. Inj Control Saf Promot.

[9] Sauter C, Zhu S, Allen S, Hargarten S, Layde PM (2005). Increased risk of death or disability in unhelmeted Wisconsin motorcyclists. WMJ.

[10] Peden M, Scurfield R, Sleet D, Mohan D, Hyder AA, Jarawan E, et al. World report on road traffic injury prevention. Geneva: World Health Organization and World Bank. Available: Uhttp://www.who.int/violence_injury_prevention/publications/road_ traffic/world_report/en/index.htmlU, accessed December 1, 2008.

[11] Government of Jamaica. Protective Device Legislation. Amendment to the Traffic Act. 1999; Sec 43D(1): 52.03.Available: www.moj.gov.jm/laws/statutes/ Road%20Traffic%20Act.pdf, accessed December 1, 2008.

[12] Crandon IW, Harding HE, Cawich SO, Frankson MAC, McLennon N, McDonald AH, et al. The Impact of Helmets on Motorcycle Head Trauma in a Developing Country. BMC Emergency Med. 2009; In Press. 10.1186/1756-0500-2-172 PMC2746805 19715612

[13] Cawich SO, Harding HE, Crandon IW, Evans NR, McDonald AH, Fearron-Boothe D. Helmet Laws in Jamaica: An Observational Study of Non-Compliant Motorcycle Accident Victims. J Forens Leg Med. 2009; In Press.

[14] International Centre for Alcohol Policies. Drinking and Driving. Module 15/ Analysis, Balance, Partnership. Available at www.icap.org, accessed January 9, 2009.

[15] National Road Safety Council: Statistics: Reports. [National Road Safety Council Website]. Available at: http://golocaljamaica.com/demo/nrsc/statistics/ reports.htm, accessed November 25 2008.

[16] Davis, A., Quimby, A., Odero, W., Gururaj, G., #& Hijar, M. Improving safety by reducing impaired driving in developing countries: A scoping study. Available: www.grsproadsafety.org/themes/default/pdfs/Impaired%20driving%20final.pdf, accessed February 2009.

[17] Moskowitz H, Burns MM, Williams AF (1985). Skills performance at low blood alcohol levels. Journal of Studies on Alcohol.

[18] Ogden EJ, Moskowitz H (2004). Effects of alcohol and other drugs on driver performance. Traffic Inj Prev.

[19] Zador PL (1991). Alcohol-related relative risk of fatal driver injuries in relation to driver age and sex. J Stud Alcohol.

[20] Zador PL, Krawchuk SA, Voas RB (2000). Alcohol-related relative risk of driver fatalities and driver involvement in fatal crashes in relation to driver update using 1996 data. J Stud Alcohol.

[21] Department of the Environment Transport and the Regions. (1998). Combating drink driving: Next steps. [Available at: www.dft.gov.uk/consultations/archive /1998/comdd/combatingdrinkdrivingnextsteps, accessed February 2009.

[22] Peek-Asa C (1999). The effect of random alcohol screening in reducing motor vehicle crash injuries. American Journal of Preventative Medicine.

[23] Simpson HM, Beirness DJ, Robertson RD, Mayhew DR, Hedlund JH (2004). Hard core drinking drivers. Traffic Inj Prev.

[24] Hedlund J (1995). Who is the persistent drinking driver? Part I: USA. Transportation Research Circular.

[25] Hingson R, Heeren T, Winter M (1998). Effects of Maine’s 0.05% legal blood alcohol level for drivers with DWI convictions. Public Health Reports.

[26] Voas RB, Blackman KO, Tippetts AS, Marques PR (2002). Evaluation of a program to motivate impaired driving. Prevention.

[27] Government of Jamaica. Child Care and Protection Act. 2004; 40: Sections 1-3. Available at: www.moj.gov.jm/laws/statutes/Child%20Care%20Protection %20Act.pdf, accessed January 9, 2009.

[28] Government of Jamaica. Spirit License Act. 1993; Section 23. Available at: www.moj.gov.jm/laws/statutes/Spirit%20License %20Act.pdf, accessed January 9, 2009.

[29] Government of Jamaica. Excise Duty Act. 1985; Section 31-39. Available at: www.moj.gov.jm/laws/statutes/Excise%Duty %20Act.pdf, accessed January 9, 2009.

[30] Government of Jamaica. General Consumption Tax Act. Available at: www.moj.gov.jm/laws/statutes/General%Consumption %Tax%20Act.pdf, accessed January 9, 2009.

[31] National Highway Traffic Safety Administration #& National Institute on Alcohol Abuse and Alcoholism. Sentencing and dispositions of youth DUI and other alcohol offenses: A guide for judges and prosecutors. Available at: http://www.nhtsa.dot.gov/people/injury/alcohol/youthdui/ index.html, accessed February, 2005.

[32] Liu B, Ivers R, Norton R, Blows S, Lo SK. Helmets for preventing injury in motorcycle riders. Cochrane Database Syst Rev. 2008, (2): CD004333. 10.1002/14651858.CD004333.pub3 PMC13092890 18254047

[33] Feldkamp G, Junghanns K (1976). Typical traffic accident in adolescents: The motorcycle accident - some epidemiologic features and the effectiveness of safety helmets and clothing. Amsterdam: Proceedings of IRCOBI.

